# Q Fever with Unusual Exposure History: A Classic Presentation of a Commonly Misdiagnosed Disease

**DOI:** 10.1155/2012/916142

**Published:** 2012-07-17

**Authors:** Randall J. Nett, Earl Book, Alicia D. Anderson

**Affiliations:** ^1^Career Epidemiology Field Officer Program, Centers for Disease Control and Prevention (CDC), Atlanta, GA 30333, USA; ^2^Communicable Disease Control and Prevention Bureau, Montana Department of Public Health and Human Services, Helena, MT 59610, USA; ^3^St. Peter's Medical Group, Helena, MT 59601, USA; ^4^Rickettsial Zoonoses Branch, CDC, Atlanta, GA 30333, USA

## Abstract

We describe the case of a man presumptively diagnosed and treated for Rocky Mountain spotted fever following exposure to multiple ticks while riding horses. The laboratory testing of acute and convalescent serum specimens led to laboratory confirmation of acute Q fever as the etiology. This case represents a potential tickborne transmission of *Coxiella burnetii* and highlights the importance of considering Q fever as a possible diagnosis following tick exposures.

## 1. Introduction

*Coxiella burnetii* is an enzootic and endemic bacterial pathogen in the United States and causative agent of Q fever [1]. Cattle, sheep, and goats are the most common animal reservoirs, and *C. burnetii* is shed in the birth products of infected animals [1, 2]. While humans typically become infected through inhalation of contaminated aerosols and dust, possible tickborne transmission of *C. burnetii* has been reported [2].

The incubation period of Q fever is usually 2–3 weeks [2]. Approximately half of persons infected with *C. burnetii* will remain asymptomatic. The most common clinical manifestation of acute Q fever is a nonspecific and febrile flu-like illness [2]; thus, the diagnosis is challenging and few patients receive appropriate treatment. Acute Q fever is treated with doxycycline and is most successful when initiated within three days of symptom onset. Approximately 1–5% of acutely ill patients will develop chronic Q fever, most commonly manifesting as endocarditis in those with valvular heart disease or immune compromising conditions [3].

*Rickettsia rickettsii* is an arthropod-borne bacterium and part of the spotted fever group rickettsiae (SFGR). The SFGR are transmitted through the bites of infected ixodid ticks [4]. *R. rickettsii* infection causes Rocky Mountain spotted fever (RMSF). RMSF generally occurs 2–14 days following infection and is characterized by fever, rash, headache, and abdominal pain [5]. Approximately 20% of patients do not develop a rash or have an atypical rash [6]. Prompt treatment with doxycycline increases the likelihood for recovery.

## 2. Case Presentation

A 57-year-old male presented on June 29, 2011 with a five-day history of fever, headache, nausea, rash, and fatigue. The patient had no history of immunosuppression. He reported exposure to multiple ticks and mosquitoes while riding horses in South Dakota in early June 2011. On examination, the patient was afebrile, diaphoretic, and had left upper quadrant tenderness. Except for documented tinea versicolor on his trunk, no other rashes were present. The remainder of the physical examination was unremarkable. Laboratory results from serum collected at the time of presentation showed a mild leukopenia, acute renal failure, elevated liver transaminases, and elevated anti-SFGR immunoglobulin (Ig)G; initial testing for Q fever was negative (Table 1). The patient was treated with doxycycline for 14 days for the presumptive diagnosis of RMSF and subsequently recovered. However, convalescent testing on July 12 revealed that the patient experienced no subsequent increase in anti-SFGR antibody, while a significant increase in phase I and II anti-*C. burnetii* IgG occurred (Table 1). The patient was judged unlikely to have been a case of RMSF as there was no demonstrable anti-SFGR IgM and no change in IgG titers. However, the patient was laboratory confirmed as infected with *C. burnetii* as demonstrated by a four-fold rise in phase II IgG-specific antibody titer between acute and convalescent specimens.

## 3. Discussion

The patient's symptom onset began approximately 2–3 weeks after exposure to multiple ticks suggesting possible tickborne disease transmission. The most common mode of human transmission is inhalational, but cases of tickborne transmission have been described [7, 8]. Over 40 tick species are naturally infected with *C. burnetii* and tick transmission to various mammals has been documented [2]. Although the patient did not report livestock contact, it is not possible to exclude this route of transmission in a natural setting because the organism can become windborne and carried for long distances [9]. Ticks from the patient or from the area of exposure were not available for testing so it is not possible to prove that *C. burnetii* infection occurred because of a tick bite rather than exposure to contaminated aerosols and dust.

The patient's initial diagnosis of RMSF was based on an elevated SFGR IgG antibody in the acute specimen. This was a reasonable assumption as the patient had a history of tick exposure, a compatible clinical presentation for RMSF and met the CDC surveillance case definition for a laboratory supportive SFGR case. However, the patient's SFGR IgG antibody did not show a significant rise in titer in the convalescent sample and no IgM antibody was detected. An acute serum specimen taken during the first week of illness is often negative as a rise in antibody titer does not typically occur until at least 7 days after symptom onset. For these reasons, it is more likely that the patient's titer to SFGR was caused by a previously undiagnosed infection rather than an acute infection as SFGR IgG response can be elevated for extended periods following acute infection [10].

This case of Q fever suggests a potential tickborne transmission of *C. burnetii* and highlights the importance of evaluating patients with suspected tickborne disease for Q fever in areas known to be endemic for *C. burnetii *[8]. Considering the diagnosis of Q fever is especially important for patients who are pregnant, immunocompromised, or have valvular heart disease as prompt antibiotic treatment can prevent life-threatening complications.

## Figures and Tables

**Table 1:** Case-patient laboratory test results, Montana, 2011.

Laboratory test	Result
White blood cell count (g/L)	4.1
Platelet count (g/L)	115
Blood urea nitrogen (mg/dL)	23
Creatinine (mg/dL)	1.7
Aspartate aminotransferase (IU/L)	132
Alanine aminotransferase (IU/L)	193
Alkaline phosphatase (IU/L)	106
*Coxiella burnetii* antibody titers^†^ by specimen	
Acute^‡^	
IgG^∗^/IgM phase I	<1 : 16/not done
IgG/IgM phase II	<1 : 16/<1 : 16
Convalescent^§^	
IgG/IgM phase I	≥1 : 256/1 : 64
IgG/IgM phase II	≥1 : 256/≥1 : 256
SFGR antibody titers^†^ by specimen	
Acute^‡^	
IgG	1 : 64
IgM	<1 : 64
Convalescent^§^	
IgG	1 : 128
IgM	<1 : 64
*Borrelia burgdorferi *antibody^¶^ (IgG and/or IgM) by specimen	
Acute^‡^	Negative
Convalescent^§^	Negative
West Nile virus antibody^¶^	
IgG	Negative
IgM	Negative

^∗^IgG: immunoglobulin G; IgM: immunoglobulin M; SFGR: spotted fever group rickettsiae.

^†^Testing performed using indirect immunofluorescence assay.

^‡^Acute specimen collected on June 29, 2011.

^§^Convalescent specimen collected on July 12, 2011.

^¶^Testing performed using enzyme-linked immunosorbent assay (ELISA); therefore, no titers reported.
